# Antitumor activity of PAbs generated by immunization with a novel HER3-targeting protein-based vaccine candidate in preclinical models

**DOI:** 10.3389/fonc.2024.1472607

**Published:** 2024-10-16

**Authors:** Ernesto Bermúdez-Abreut, Gretchen Bergado Báez, Melissa Martínez Pestano, Giuseppe Attanasio, Carlos Yordan Gonzales Castillo, Diana Rosa Hernández Fernández, Rydell Alvarez-Arzola, Andrea Alimonti, Belinda Sánchez-Ramírez

**Affiliations:** ^1^ Immunology and Immunotherapy Division, Center of Molecular Immunology (CIM), Havana, Cuba; ^2^ Department of Molecular Oncology, Institute of Oncology Research (IOR), Bellinzona, Switzerland; ^3^ Faculty of Medicine, Università della Svizzera Italiana, Lugano, Switzerland; ^4^ Department of Medicine, University of Padua, Padua, Italy; ^5^ Medical Oncology, Oncology Institute of Southern Switzerland, Bellinzona, Switzerland

**Keywords:** HER3, ErbB3, immunotherapy, cancer vaccines, PAbs, antitumor effect

## Abstract

Despite the cumulative evidence supporting HER3 as a target for antitumor therapies, no agents targeting HER3 have been approved for cancer treatment. Most of the agents evaluated in preclinical and clinical trials have been specific monoclonal antibodies (MAbs), with few examples of active immunotherapy directed against this receptor. However, some cancer vaccine formats may generate polyclonal antibodies (PAbs) that replicate the diverse effector mechanisms of MAbs, including ligand neutralization and receptor degradation. In this study, we developed a protein subunit-based monovalent vaccine candidate targeting the extracellular domain (ECD) of HER3. Immunization of mice with a formulation targeting murine ErbB3-ECD successfully overcome tolerance to this self-antigen, inducing high titers of ErbB3-specific PAbs. The antitumor potential of this formulation and the induced PAbs was demonstrated *in vivo* and *in vitro* in an ErbB3-overexpressing 3LL-D122-derived tumor model. The immunogenicity of the HER3-ECD-based vaccine candidate was confirmed by the induction of high titers of HER3-specific PAbs. Consistent with the initial results, HER3-ECD-targeting PAbs were cytotoxic in several human epithelial tumor cell lines and exerted antitumor effects *in vivo*. These results support the value of HER3 as a tumor antigen and the effector mechanisms of HER3-specific therapeutic MAbs, while suggesting the potential of the proposed vaccine candidate for the treatment of HER3-expressing carcinomas.

## 1 Introduction

Human epidermal growth factor receptors (HER) are among the most studied tyrosine kinase receptors that contribute to oncogenesis and tumor progression, consistent with the definition of EGFR (HER1) and HER2 as relevant tumor antigens (1, 2). It is extensively described that the upregulation or aberrant expression of HER family members in epithelial tumors correlates with rapid progression of the disease and lower patient survival (3).

Due to its minimal kinase activity, HER3 was largely underrated as a target for antitumor therapies. Nevertheless, in the last 20 years, cumulative evidence has demonstrated the importance of HER3 in cancer progression, increased invasion, and acquired drug resistance in epithelial tumors of different locations (4, 5). For example, HER2–HER3 signaling is highlighted as a relevant oncogenic driver in breast carcinomas that overexpress HER2. In this scenario, tumor progression is abrogated through shRNA or CRISPR Cas-mediated HER3 silencing (6, 7). Other studies have reported an important role of HER3 in non-small cell lung cancer (NSCLC) and, more recently, in prostate cancer evolution (8–10). Also, HER3 activation has been shown to predict resistance to antitumor therapies, including chemotherapy, HER1-targeting inhibitors, estrogen antagonists, or radiation (9, 11–13). In patients, HER3 overexpression is crucial in the progression of human cancers and is associated with poorer overall survival across many solid tumors (4, 14, 15). Additionally, some reports suggest that HER3 activation is a major cause of treatment failure and is also considered a resistance mechanism in cancer therapy in different types of tumors (8, 16–20). All of these factors emphasize the importance of developing an effective HER3 targeting strategy.

Although HER3 expression has been observed in various nonmalignant tissues, similar to other members of the EGFR family (HER1 and HER2), which have different approved treatments for different types of cancer, early clinical trials with agents targeting HER3 have shown a favorable toxicity profile (1), indicating the safety of these HER3 treatments. Since this receptor lacks tyrosine kinase activity (21), it cannot be specifically targeted with the tyrosine kinase inhibitors that have been successful against HER1 and HER2 (1). As a result, the primary strategy has been to develop monoclonal antibodies aimed at blocking this receptor.

While several MAbs targeting the extracellular portion (domain) of HER3 have been tested in preclinical studies and phase I/II clinical trials for therapeutic use in oncology, only three of these antibodies (patritumab, seribantumab, and lumretuzumab) have shown limited improvement in patient outcomes. These are currently in advanced clinical trials, but only a few objective responses have been observed, suggesting limited activity as single agents (1, 22–24). Intrinsic factors, such as tumor heterogeneity and mutations in the recognition epitope of the antibody, may contribute to the limited efficacy of HER3-targeting MAbs. This indicates that the antitumor effects of these agents could be surpassed by alternative approaches. For instance, a more extensive inhibition and downregulation of HER3 could be achieved through the recognition of nonoverlapping epitopes by MAbs, which may be optimally achieved with polyclonal antibodies (PAbs) induced by vaccination (25). Considering the benefits of active immunotherapy and studies suggesting the antitumoral advantages of a polyclonal response over monoclonal antibodies (25–27), developing a vaccine against HER3 could be a strategy for treating cancers in which the activation of this receptor plays a fundamental role.

This work presents a new vaccine candidate that comprises the extracellular domain (ECD) of HER3 (HER3-ECD) as an antigen vaccine. Taking as a reference the current formulation of other vaccine candidates reported by our group, the monovalent HER3-based vaccine candidate (Mv-HER3) includes a combination of Montanide ISA 51 VG and very small size particles (VSSP) as adjuvants (28–30). Herein, we assessed the ability of the autologous murine ErbB3-based formulation to overcome tolerance to self-antigens and elicit an antitumor effect. Consistent with this, immunization of mice with the Mv-HER3 induces anti-HER3 PAbs that impair cell viability *in vitro* or tumor growth *in vivo* in HER3-positive epithelial tumor cell lines. This effect may be related to the vaccine’s ability to downregulate HER3 and probably other physically interacting oncogenic receptors (like HER1 and HER2), suggesting opportunities for therapeutic combinations.

## 2 Materials and methods

### 2.1 Cell lines and culture conditions

Three prostate human cell lines—PC3 (CRL-1435), DU 145 (HTB-81), and LN-Cap (CRL-1740)—along with the human cell lines derived from NSCLC, H1975 (CRL-5908), and SKBR3 (HTB-30, isolated from a breast adenocarcinoma), were obtained from the American Type Tissue Culture Collection (ATCC). The PC9 cell line and erlotinib-resistant PC9ER cell line were obtained from the Department of Biological Regulation at the Weizmann Institute of Sciences (Israel). The 3LL-R3 (3LL-D122 derived) tumor line was previously generated at the Center of Molecular Immunology. This model is resistant to the 7A7 MAb specific to murine EGFR, partially due to the upregulation of murine ErbB3 (mErbB3) compared to the parental 3LL-D122 line (31). All above-mentioned cell lines (except SKBR3) were maintained in basal growth media RPMI (Gibco, USA), supplemented with 10% fetal bovine serum (FBS; Gibco) and penicillin/streptomycin (100 µg/mL). SKBR3 cells were grown in McCoy’s 5A culture medium (Gibco), supplemented with 15% FBS. All cells were grown at 37°C in a humidified atmosphere containing 5% CO_2_.

### 2.2 Recombinant proteins

The extracellular domains of the murine and human variants of the ErbB3 (mErbB3 and HER3) receptor were obtained as recombinant proteins through transient transfection into HEK293T cells. (see **Supplementary Figure S1** for more details on mErbB3-ECD and HER3-ECD generation). The HER1-ECD and HER2-ECD were generated at the Immunology and Immunotherapy Direction of the Center of Molecular Immunology (CIM, Cuba), along with the four subdomains (I, II, III, and IV) included in HER3-ECD and the irrelevant control PDL1 fused to His-tag.

### 2.3 ELISA assays

Microtiter plates (High Binding, Costar, USA) were coated with 10 μg/mL of different recombinant proteins in carbonate buffer (0.1 M, pH 9.6) and incubated overnight at 4°C (for antibody titration, mErbB3-ECD-His or HER3-ECD-His were used; for determining antibody specificity, HER1-ECD, HER2-ECD, HER3-ECD-His, and PDL1-His recombinant proteins were used; for polyclonal response determination, the four subdomains [I, II, III, and IV] of the HER3-ECD recombinant proteins were used). Subsequently, plates were washed with 0.05% Tween 20 in phosphate-buffered saline (PBS; washing buffer) and blocked for 1 h at room temperature with assay buffer (4% bovine serum albumin and 0.5% Tween 20 in PBS). Sera (immune or preimmune) dilutions in assay buffer were incubated for 1 h at 37°C, followed by incubation with HRP-conjugated goat antimouse IgG antibody (71045, Sigma, USA) for 1 h at 37°C. Finally, orto-phenylendiamine (OPD) peroxidase substrate (Sigma) was added, and the plates were incubated in the dark for 30 min at room temperature (RT). The reaction was stopped using 10 M H_2_SO_4_. The optical density (OD) at 490 nm was measured using a microwell reader (Organon Teknica, Salzburg, Austria). All incubations were followed by three washing steps with washing buffer. An OD value of at least twice the value of the corresponding control was established as a criterion to consider a positive signal in the test. All samples were evaluated in triplicate.

### 2.4 Western blot assays

Cells were grown to 70% confluence in six-well plates (Greiner, USA) and treated with anti-HER3 sera or preimmune sera diluted 1:100 in serum-free culture medium for 24 h. The cells were then washed twice with ice-cold PBS and scraped into lysis buffer (50 mM HEPES [pH 7.5], 10% glycerol, 150 mM NaCl, 1% Triton X-100, 1 mM EDTA,1 mM EGTA, 10 mM NaF, 0.1 mM Na_3_VO_4_, and a complete protease inhibitor cocktail). Next, lysates were centrifuged at 14,000×*g* for 15 min at 4°C, and the supernatants were collected for further procedures. Proteins (50 µg) were separated using gel electrophoresis and transferred to nitrocellulose membranes. After blocking, membranes were incubated overnight with primary antibodies: anti-HER3 (AF234, R&D Systems, USA), anti-HER2 (AF1129, R&D Systems), anti-HER1 (AF231, R&D Systems), or ß-actin (MAB8929, R&D Systems). This was followed by incubation with horseradish peroxidase-conjugated secondary antibodies for 1 h and treatment with Clarity™ Western ECL Blotting Substrates (Bio-Rad). ECL signals were detected using the ChemiDoc™ Imaging System (Bio-Rad, USA), and images were acquired with ImageLab software.

### 2.5 Flow cytometry assays

#### 2.5.1 Recognition of tumor cells by immune sera from mice immunized with the Mv-HER3

3LL-R3 cells were stained with anti-mErbB3 sera or with anti-HER3 sera. SKBR3, DU145, PC9ER, LN-Cap, and PC3 cells were stained with anti-HER3 sera (sera diluted 1:200) for 20 min at 4°C. Afterward, cells were incubated with FITC-linked goat antimouse IgG (Sigma, 1:100) for 20 min at 4°C. Five thousand events were acquired in a Gallios Cytometer (Beckman Coulter, San Jose, CA, USA), and data were processed with FlowJo 10 software.

#### 2.5.2 Evaluation of apoptosis markers

H1975 cells were seeded in 12-well plates (0.2 × 10^5^ cells/well) and maintained in their culture conditions for 24 h. Subsequently, the supernatant was removed, and the cells were treated with mixtures of the immune sera (1/20), which had been previously inactivated to neutralize the contribution of complement (by incubation at 56°C for 30 min). Untreated cells and cells treated with a mixture of PI sera were used as negative controls. MitC (5 μg/mL) was used as a positive control for apoptosis induction in all assays. Treatments were prepared in RPMI medium supplemented with 1% SFT. After 48 h of treatment under the described conditions, the cells were acquired by trypsin digestion and washed with SSTF by centrifugation at 300×*g* for 5 min. Subsequently, labeling was performed to detect the molecular markers of interest.

A TUNEL reagent set was used to detect DNA fragmentation, following the manufacturer’s recommendations. Cells were fixed with 0.25% paraformaldehyde for 1 h at 25°C, after which they were washed twice by centrifugation at 300×*g* for 10 min. They were then permeabilized with a solution containing 0.1% (m:v) sodium citrate and 0.1% (v:v) Triton X-100 for 2 min at 4°C. Permeabilized cells were washed twice under the previously described conditions and incubated then with a mixture of the enzyme terminal deoxynucleotidyl transferase and FITC-conjugated dUTP nucleotides (1:9 [v:v]) for 1 h at 37°C in the dark.

To detect the presence of the enzyme caspase 3 (in its active form) in the treated cells, a MAb specific for a fragment resulting from the proteolytic activation of this enzyme, conjugated with the Alexa-Fluor^®^488 fluorophore, was used. Cell labeling was performed according to the manufacturer’s instructions. Cells were fixed and permeabilized with Cytofix/Cytoperm solution included in the Golgi Stop Reagent Set (R&D Systems, USA) for 25 min at 4°C. Subsequently, the cells were incubated with the antiactive caspase 3 MAb conjugated with Alexa Fluor^®^488 (1:50), diluted in perm/wash solution of this set of reagents, for 30 min at 4°C. Between the aforementioned incubations, the cells were washed twice with the perm/wash solution diluted 10 times in distilled water by centrifugation at 300×*g* for 5 min.

For both markers, 5 × 10^3^ cells were acquired on a FlowSpace flow cytometer (PARTEC), and the data obtained were analyzed using FlowJo version 10 software.

### 2.6 MTT assays

Cell viability was assessed by MTT (No. M5655, Sigma-Aldrich). Cells were plated in 96-well plates at the following densities: 5 × 10^3^ cells/well for the 3LLR3, SKBR3, DU145, H1975, LN-Cap, and PC3 cell lines, and 3 × 10^3^ cells/well for the PC9 and PC9ER cell lines. When all cells reached 60%–70% confluency, growth media were removed, and the cells were incubated with anti-mErbB3 or anti-HER3 immune sera (diluted 1:20 in growth media, supplemented with 1% FBS). Prior to their addition to the cells, the sera were incubated at 56°C for 30 min to inactivate the complement. Cells treated with preimmune sera (preheated and diluted as described) were considered negative controls for the assay. After 96 h, the MTT reagent was added to the cells (1 mg/mL), and 2 h later, the formazan crystals were dissolved in DMSO. Absorbance was measured at 540 nm, with background absorbance at 630 nm subtracted. Untreated cells were considered the maximum viability control. Tyrosine kinase inhibitors (TKI) specific to wild-type or mutant HER1 were used as positive controls for assessing cell viability decrease associated with HER receptor inhibition: AG1478 (No. T4182, Sigma-Aldrich) at 10 µM for SKBR3 and PC9 cells, and osimertinib (Tagrisso, AstraZeneca, USA) at 1 µM for PC9ER and H1975 cells. Doxorubicin (D1515, Sigma-Aldrich) was used as a control for general cytotoxicity induction (10 µM). Treated cell viability percentage was calculated using the following formula: cell viability (%) = (OD treated cell_540–630 nm_)/(OD nontreated cell_540–630 nm_) × 100. All samples were evaluated in quintuplicates.

### 2.7 Animals

Female C57BL/6 mice aged 8–12 weeks old, female Nu/Nu nude mice aged 6 weeks old, and male New Zealand (NZD) rabbits were acquired from the National Center for Laboratory Animals Production (CENPALAB, Havana, Cuba). Mice and rabbits were kept under pathogen-free conditions. All animal experiments were approved by the Center of Molecular Immunology’s Institutional Animal Care and Use Committee (CIM, Havana, Cuba).

### 2.8 Immunization protocols

C57/BL6 mice were immunized four times, spaced biweekly, with 200 μg of mErbB3-ECD-His or HER3-ECD-His. Both preparations were adjuvanted in 200 μg of VSSP per mouse and emulsified with Montanide ISA 51 VG. Sera were obtained from blood extractions performed on days − 2 (preimmune serum), 7, 21, 35, and 49.

Alternatively, male NZD rabbits were immunized four times as previously described to stimulate HER3-specific PAbs production. On day 49, both immunized and nonimmunized rabbits were bled, and sera were extracted from blood and subsequently dialyzed in a 0.02-M sodium acetate buffer containing 0.2 M of sodium chloride. Next, the whole fraction of IgG isotype PAbs was purified by Protein A chromatography. Irrelevant PAbs were obtained from a nonimmunized NZD rabbit using the same methodology.

### 2.9 Antitumor effect of the mErbB3/HER3 vaccine in immunocompetent mice

C57/BL6 mice were divided into two groups, with four mice in each group, to assess the mErbB3-ECD antigen. The antitumor properties of the Mv-HER3 vaccine were also studied in C57/BL6 mice, divided into two groups, with five mice in each. In both experiments, the first group of mice was immunized with the mErbB3-ECD antigen or the Mv-HER3 vaccine as described above. The second group in both experiments did not receive any treatment during the immunization schedule. Seven days after the last immunization, all mice were intravenously injected with 50,000 3LL-R3 cells. Three weeks after tumor inoculation, the mice were weighed and killed. Subsequently, the lungs were removed and weighed as an indicator of metastatic burden.

### 2.10 Antitumor effect of anti-HER3 PAbs in nude mice

DU145 cells (3 × 10^6^ cells per mouse diluted in 100 µL of PBS) were subcutaneously injected into the right flanks of 8-week-old female Nu/Nu nude mice (NU-*Foxn1^nu^*). Once the tumors became detectable, the mice were randomized into two groups of four. One group received five intraperitoneal administrations of vaccination-induced PAbs (1 mg IgG/mouse/injection). A second (control) group of mice was equally treated with irrelevant PAbs. Tumors were measured with a caliper. At the experimental endpoint, all mice were killed, and their tumors were removed and weighed. Tumor volume (*T*_volume_) was calculated using the formula: *T*_volume_ (mm^3^) = *L*_1_/2*L_2_/2*H*4/3*π; where *L*_1_ and *L*_2_ are two measurements of tumor diameters, and *H* is the height of the tumor from the mouse skin.

### 2.11 Statistical and data analyses

GraphPad Prism 7.0 software was used for statistical analysis. Normality was evaluated using the Shapiro–Wilk test, and variance homogeneity was analyzed with either Levene’s test or the Brown–Forsythe test. Statistical differences between the media of two groups were analyzed using a *t*-test, while statistical differences among the media of more than two groups were analyzed using one-way ANOVA followed by the Tukey’s multiple comparisons test. Statistical differences among tumoral kinetics curves were analyzed using two-way ANOVA. Statistical power was analyzed using the PASS 15.0.1 program, with results showing more than 85% statistical power for all corresponding cases. In graphic representations, significant differences were highlighted with asterisks: ^*^ *p* < 0.05, ^**^ *p* < 0.01, ^***^ *p* < 0.001, and ^****^ *p* < 0.0001.

## 3 Results

### 3.1 Immunization of mice with self mErbB3 elicits antitumor effect in a metastasis setting

**Figure 1:**
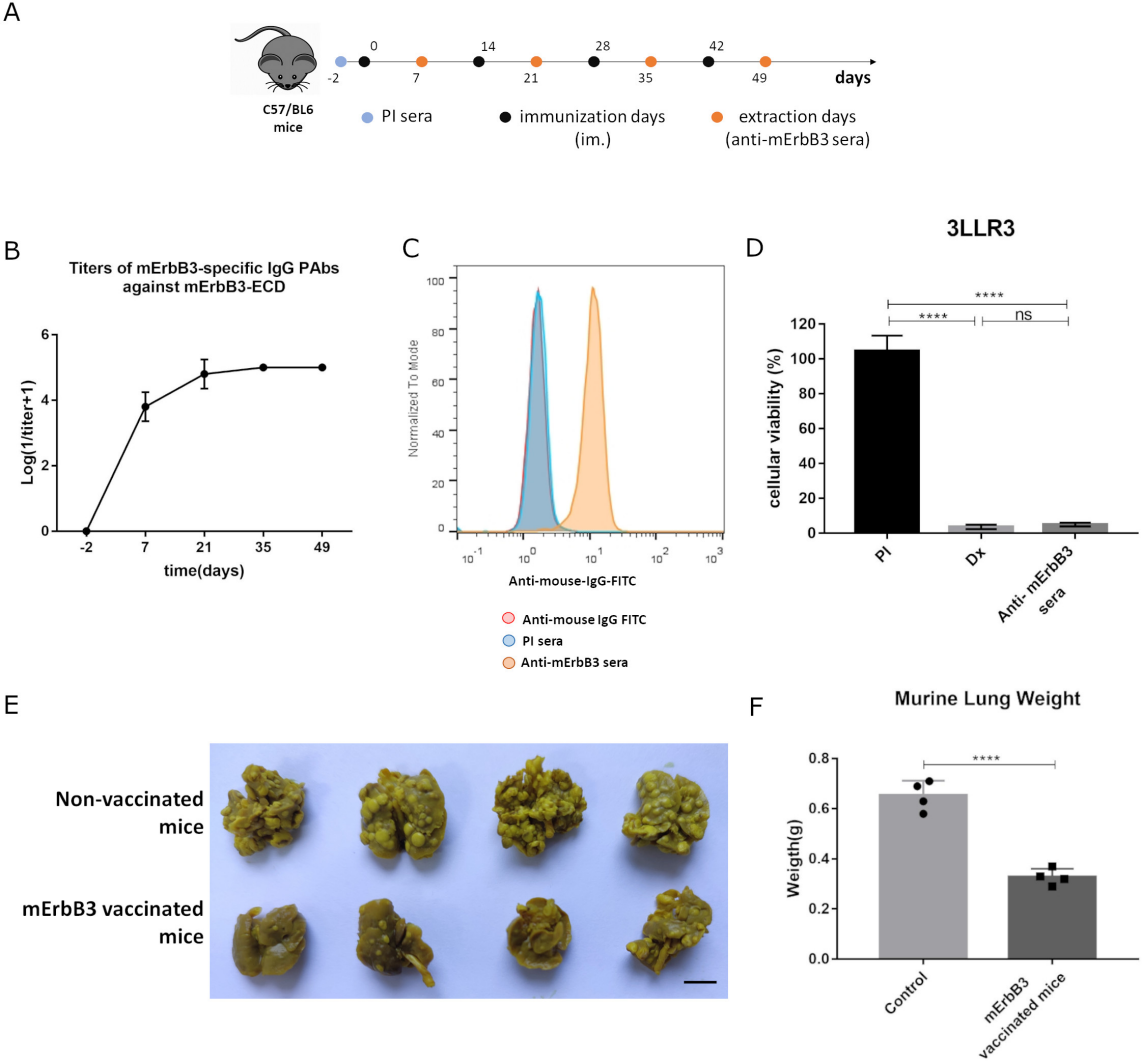
PAbs response and antitumor effect following immunization with a murine ErbB3-ECD-specific vaccine formulation. **(A)** C57BL/6 mice were immunized with murine ErbB3-ECD (200 μg) adjuvanted in VSSP (200 μg)/Montanide (intramuscularly (im.), 50 μL). Four doses were administered every 14 days. On specified days, blood samples were drawn and **(B)** induced anti-mErbB3 PAbs were titrated by ELISA. **(C)** Recognition of mErbB3 on 3LL-R3 tumor cells by the PAbs present in the immune sera (1:200, yellow histograms) was analyzed by flow cytometry. Cells incubated with preimmune sera (PI, blue histograms) or stained directly with the conjugated antibody (red histograms) served as controls. **(D)** 3LL-R3 cells were treated with immune sera (heat-inactivated and 1:20 diluted). After 96 h, cell viability was determined using MTT assay. PI sera and doxorubicin (Dx, 10 μM) were included as negative and positive controls, respectively. Differences among treatment means in a representative experiment were analyzed using one-way ANOVA, and Tukey’s was used for multiple comparisons. **(E)** Mice immunized as described above were challenged intravenously with 50,000 3LL-R3 cells. The image displays lungs extracted from immunized or control mice 3 weeks later. Scale bar, 1 cm. **(F)** Media ± SD of tumor weight of four animals per group is shown and compared using a *t*-test. Significant differences are represented as ^****^ *p* < 0.0001. All results shown are representative of at least two experiments performed individually.

In a four-dose induction scheme (**Figure 1A**), immunization with murine ErbB3-ECD (mErbB3) (adjuvated in VSSP/Montanide) induces a specific PAbs response. As observed (**Figure 1B**), the antibody titers reached a maximum of 1:10^5^ for all mice after the third immunization and remained at the same level after the fourth dose. These results suggest the ability of this formulation to circumvent tolerance to self-antigens, as high antibody titers are induced. These anti-mErbB3 PAbs contained in the sera of vaccinated mice recognized the mErbB3 receptor (in its native conformation) expressed in 3LL-R3 tumor cells. This effect was not reproduced by preimmune sera, which confirms the specificity of this result (**Figure 1C**). 3LL-R3 cells treated with anti-mErbB3 PAbs induced a reduction in cell viability compared to the preimmune sera control (4.98% ± 1.08% vs. 104.7% ± 8.79%, *p* < 0.0001). No statistically significant difference was found between the reduction in viability caused by anti-mErbB3 sera and the doxorubicin control (**Figure 1D**). Furthermore, vaccination with the murine ErbB3-ECD-based vaccine candidate inhibited 3LL-R3 tumor progression in an experimental metastasis setting, as fewer metastases were observed in the lungs of vaccinated mice compared to untreated controls (**Figure 1E**), resulting in lower lung weight (0.33 g ± 0.03 g vs. 0.65 g ± 0.06 g, *p* < 0.0001) (**Figure 1F**).

### 3.2 HER3-based vaccine candidate (Mv-HER3) induces a specific polyclonal response

**Figure 2:**
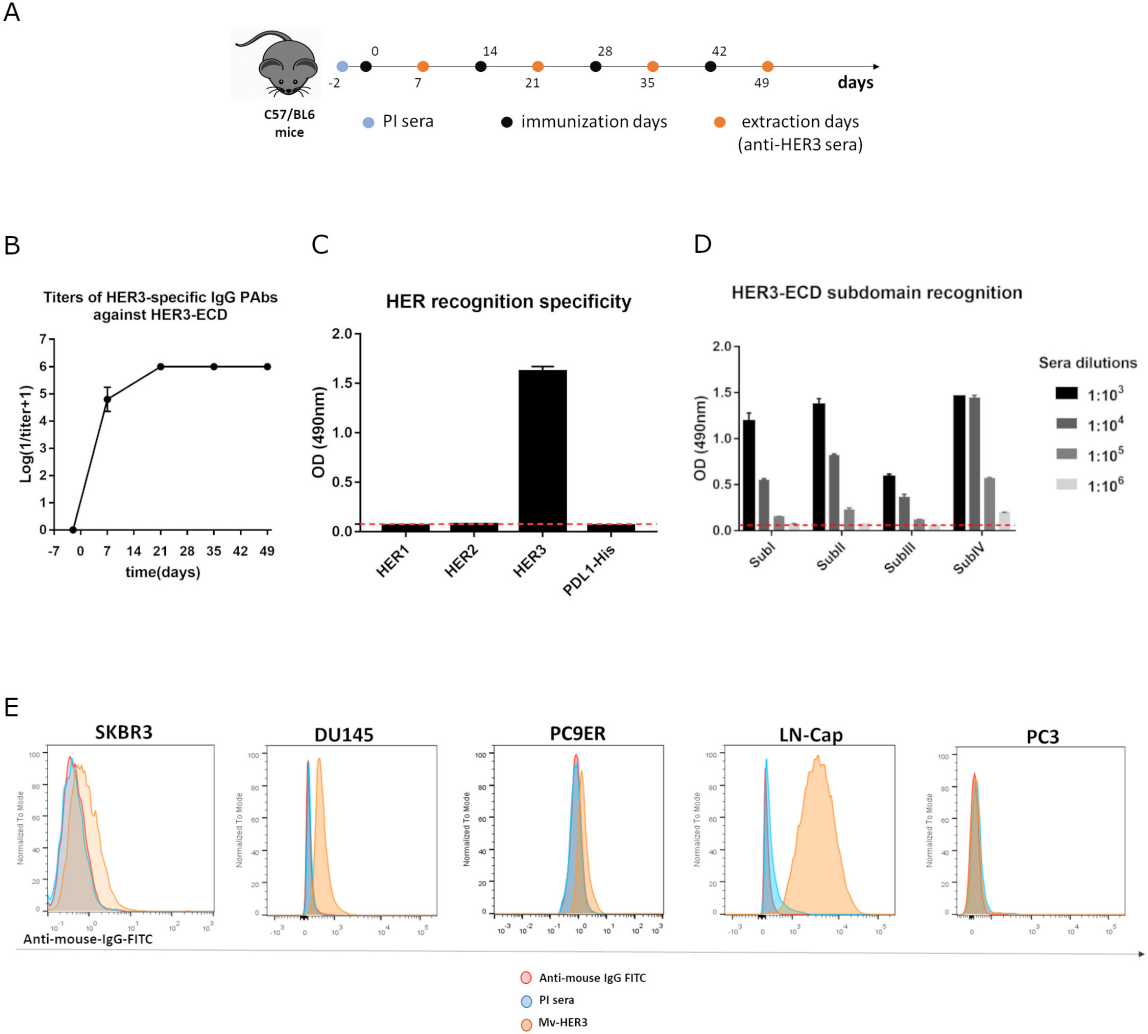
Polyclonal antibody response of Mv-HER3. **(A)** C57BL/6 mice were immunized with HER3-ECD (200 μg) adjuvanted in VSSP (200 μg)/Montanide (intramuscularly (im.), 50 μL). Four doses were administered every 14 days. Blood samples were drawn in specified days, and **(B)** anti-HER3-induced PAbs were titrated by ELISA. **(C)** Recognition of related HER receptors and a His-tag-irrelevant protein (PDL1-His) by immune sera (diluted 1:1000) was evaluated using ELISA. The preimmune (PI) sera (dotted red line) served as an additional specificity control. **(D)** Recognition of the four subdomains of HER3-ECD by immune sera was determined by ELISA. In the graphs, the dotted red line represents the average of optical density against all four subdomains for the PI sera. **(E)** Recognition of HER3-positive (SKBR3, DU145, PC9ER, LNCap) and HER3-negative (PC3, negative control) human tumor cells by immune sera (1:200, yellow histograms) was determined by flow cytometry. Cells incubated with the preimmune sera (PI, blue histograms) or stained directly with the conjugate (red histograms) served as technical controls. All results shown are representative of at least two experiments performed individually.

Avoiding tolerance has been one of the greatest challenges in vaccinology, particularly concerning autoantigens. In this context, the results previously obtained with the autologous mErbB3 suggested the ability to circumvent tolerance and the therapeutic potential of a vaccine candidate that included the extracellular domain of the human ErbB3 (HER3) in patients. Considering the formulation and immunization scheme previously described, the immunogenicity of the Mv-HER3 was studied in mice (**Figure 2A**). After the first dose, PAbs titers oscillated between 1:10^4^–1:10^5^. Maximum titer (1:10^6^) was reached right after the second dose and remained stable afterward for all vaccinated mice (**Figure 2B**). Of note, PAbs titers against this human variant (HER3-ECD) were higher (an order of magnitude) if compared with the titers achieved by immunization against the murine variant (see **Figure 1B**), probably because the HER3-ECD is a nonself-antigen in mice. As shown in **Figure 2C**, the PAbs response induced by Mv-HER3 was specific against HER3-ECD, as immune sera did not recognize the ECD of related receptors HER1 and HER2 (**Figure 2C**). Additionally, immune sera reacted against all four subdomains of HER3-ECD in a dose-dependent manner, which confirms the polyclonality of the humoral response induced by the Mv-HER3 (**Figure 2D**). As part of the study of the polyclonal response triggered by the vaccine candidate, IgG subclasses in the HER3-specific PAbs induced in vaccinated mice were examined (see **Supplementary Figure S2**). Elevated levels of IgG1, IgG2a, and IgG2b were observed in the sera obtained after the four immunization doses (day 49), suggesting a mixed pattern of Th1/Th2 response. Finally, in a panel of different HER3-positive human tumor cell lines (see **Supplementary Figure S3**), the anti-HER3 PAbs present in the immune sera specifically recognize the HER3 molecule (**Figure 2E**).

### 3.3 Antitumor effect of vaccination with Mv-HER3 in 3LL-R3 murine model

**Figure 3:**
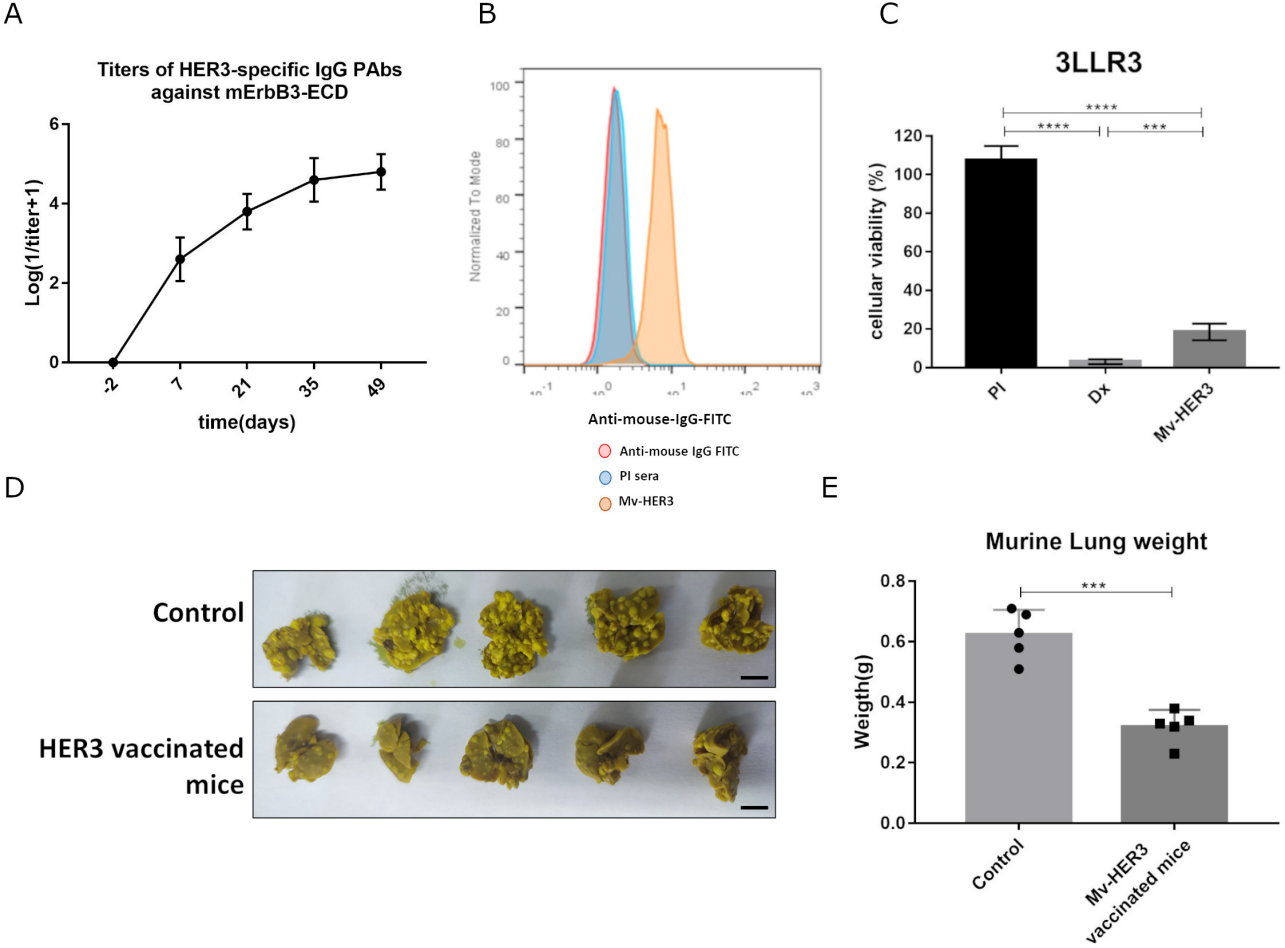
Antitumor effect of vaccination with the HER3-based vaccine candidate in the murine 3LL-R3 tumor model. **(A)** Anti-HER3 PAbs induced were titrated using ELISA against mErbB3-ECD. **(B)** Recognition of mErbB3 on 3LL-R3 tumor cells by anti-HER3 PAbs present in the immune sera (1:200, yellow histograms) was analyzed by flow cytometry. Cells incubated with preimmune sera (PI, blue histograms) or stained directly with the conjugate (red histograms) served as controls. **(C)** 3LL-R3 cells were treated with immune sera (heat inactivated and 1:20 diluted). After 96 h, cell viability was determined using the MTT assay. PI sera and doxorubicin (Dx, 10 μM) served as negative and positive controls, respectively. Differences among treatment means in a representative experiment were analyzed by one-way ANOVA, with Tukey’s test applied for multiple comparisons. **(D)** Mice immunized as described were challenged intravenously with 50,000 3LL-R3 cells. The image displays lungs extracted from immunized or control mice 3 weeks later. Scale bar, 1 cm. **(E)** Media ± SD of tumor weights of five animals per group is shown and compared using a *t*-test. Significant differences are represented as ^***^ *p* < 0.001 and ^****^ *p* < 0.0001. All results shown are representative of at least two experiments performed individually.

On the other hand, we calculated the percentage of sequence identity between the extracellular domains of the human and murine variants of ErbB3 using the online tool NCBI Blast: Protein Sequence (nih.gov). Considering that we found 92.7% homology between these sequences, we evaluated whether the antibodies generated with the Mv-HER3 candidate were capable of recognizing the murine ErbB3 receptor. The PAbs titers in sera of mice immunized with the Mv-HER3 candidate against the murine variant of the mErbB3 receptor were between 1:10^4^ and 1:10^5^ and suggest the strong recognition of this PAbs for the murine receptor ErbB3 (**Figure 3A**). It was also found that PAbs generated by the Mv-HER3 candidate were capable of recognizing the murine ErbB3 receptor on the cell membrane surface (**Figure 3B**) and induced a cytotoxic effect in the 3LL-R3 tumor cell line compared with the PI control (18.48% ± 4.29% vs. 107.5% ± 7.40%, *p* < 0.0001) (**Figure 3C**). Considering all these results, we then evaluated the antitumor effect of vaccination with the Mv-HER3 candidate in the murine 3LL-R3 tumor cell line. In this context, we found that vaccination with the Mv-HER3 candidate inhibited murine 3LL-R3 tumor progression in an experimental metastasis setting, as fewer metastases were observed in the lungs of vaccinated mice compared to unvaccinated mice (**Figure 3D**). Consequently, Mv-HER3-vaccinated mice exhibited lower lung weight compared to control mice (0.32g ± 0.06 g vs. 0.62g ± 0.08 g, *p* < 0.0001) (**Figure 3E**).

The high sequence identity between the human and murine ErbB3 variants also makes the murine model a relevant species to evaluate the potential toxicity of the HER3-based vaccine candidate. To evaluate this, a control group and a group receiving the Mv-HER3 candidate vaccine were compared. The mice’s weight was measured after each immunization, and after the vaccination protocol was completed, the animals were killed to analyze signs of toxicity in organs such as the heart, kidney, and liver. No visible adverse effects or signs of toxicity were observed in the behavior, weight, or histology of the analyzed organs, indicating the safety of the HER3-based vaccine candidate (see **Supplementary Figure S4**).

### 3.4 Mv-HER3-induced PAbs promote HER3 degradation and induce cytotoxicity in human tumor models

**Figure 4:**
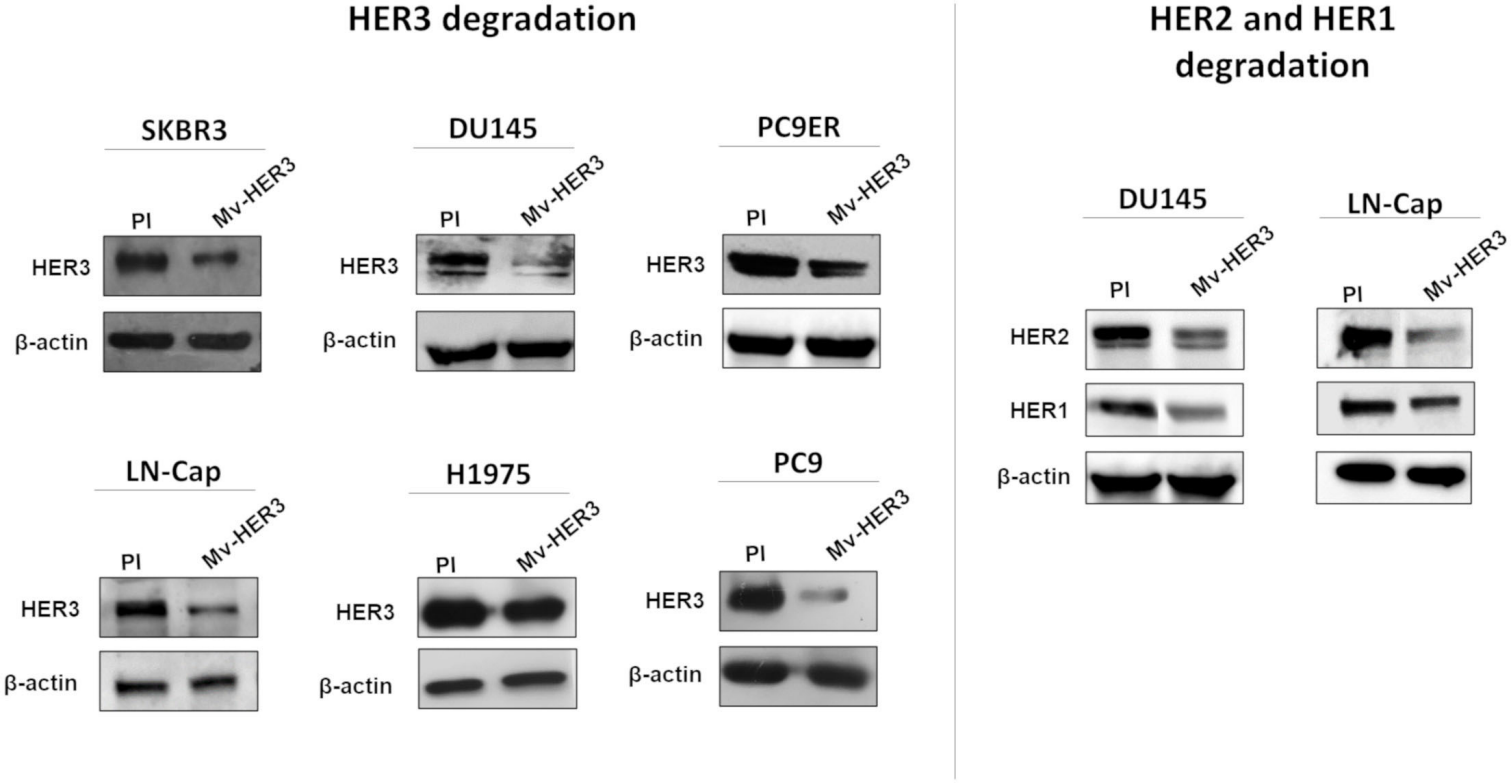
HER receptor degradation was promoted by the sera obtained from mice vaccinated with the Mv-HER3 candidate. SKBR3, DU145, PC9ER, LNCap, H1975, and PC9 cell lines were incubated for 24 h with a mixture of anti-HER3 sera (1:100 diluted). Cells treated with a mixture of preimmune (PI) sera (1:100 diluted) served as a control. Protein expression of HER3 was determined by Western blotting (left panel). In these experiments, β-actin was used as a loading control. In DU145 and LNCap cells, HER2 and HER1 expression was also analyzed by Western blotting after 24 h of treatment with immune sera (1:100 diluted) (right panel). The images display autoradiography films corresponding to the detection of the molecules above mentioned in one representative experiment of two conducted for each cell line.

Next, the potential antitumor properties of the Mv-HER3 candidate were evaluated in human tumor cells. First, the capacity of Mv-HER3-induced PAbs to downregulate HER3 receptor following recognition in human tumor cells was assessed (**Figure 4**). As shown in **Figure 4**, treatment with immune sera resulted in decreased HER3 protein levels compared to preimmune sera-treated cells in all evaluated tumor human cell lines, indicating degradation of the HER3 receptor. Using DU145 and LN-Cap tumor cells as models, it was evident that sera containing anti-HER3 PAbs could also promote the degradation of HER1 and HER2. Since the specificity of Mv-HER3-induced PAbs had already been demonstrated, their ability to promote HER1 and HER2 degradation suggests the capacity of anti-HER3 PAbs to downregulate interacting receptors in the context of heterodimers.

**Figure 5:**
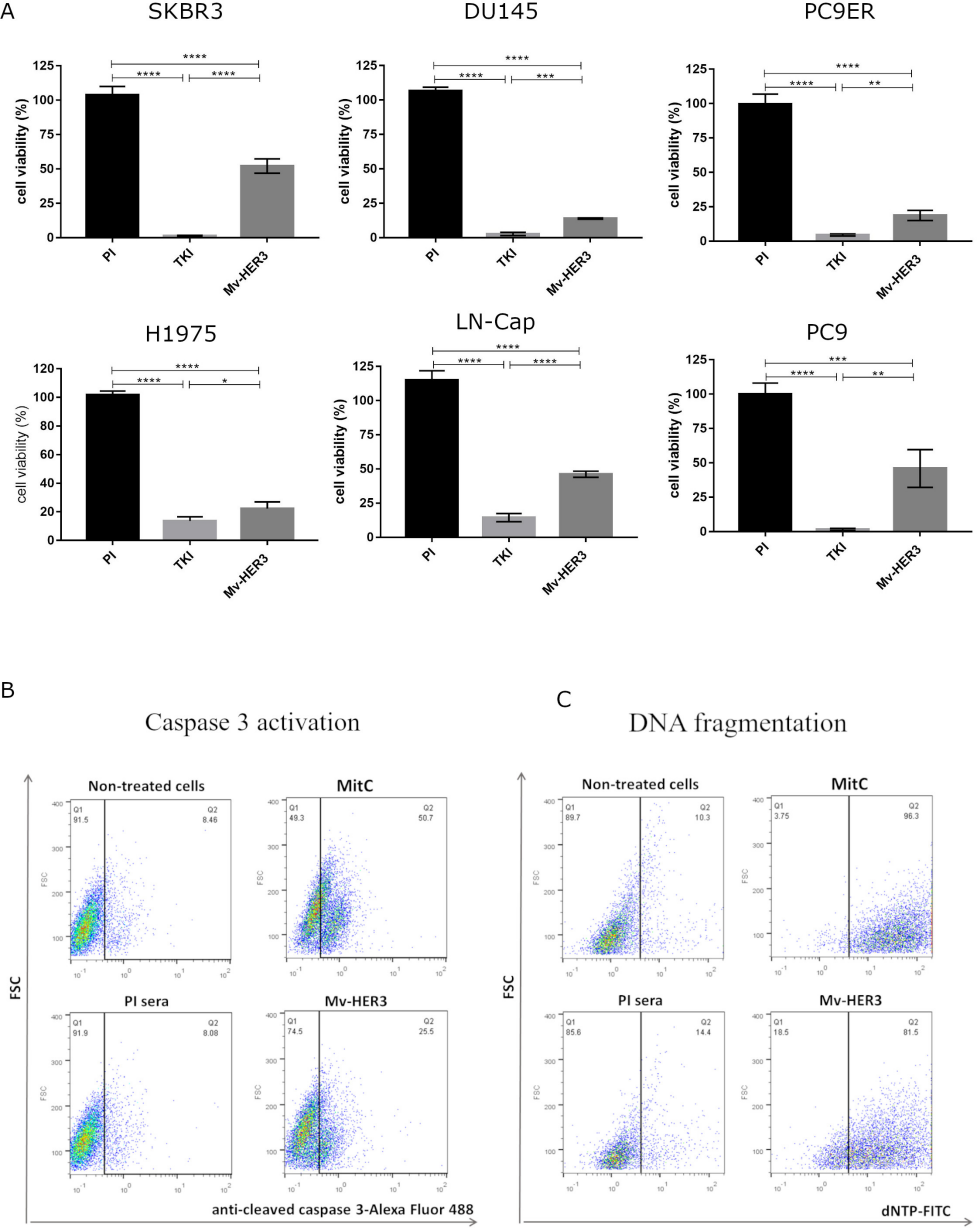
Inhibition of cell viability in a panel of human tumor cell lines treated with sera obtained from mice immunized with the Mv-HER3 candidate. **(A)** Cells from the SKBR3, DU145, PC9ER, H1975, LNCap, and PC9 cell lines were treated for 96 h with a mixture of sera obtained from mice immunized with the Mv-HER3 vaccine candidate (heat-inactivated and diluted 1:20). Cell viability was quantified using the MTT assay. Preimmune (PI) sera served as a negative control. TKI specific for wild-type (AG1478) or T790M-mutant HER1 (osimertinib) were used as positive controls of cytotoxicity induction as follows: 10 µM of AG1478 (for SKBR3, DU145, LN-Cap, and PC9 cells) or 1 µM osimertinib (for PC9ER and H1975 cells). The graphs represent one experiment that is representative of at least two conducted for each tumor cell line. Differences among means were analyzed using one-way ANOVA, with the Tukey’s test applied for multiple comparisons. Significant differences among treatments are represented as ^*^ *p* < 0.05; ^**^ *p* < 0.01; ^***^ *p* < 0.001; ^****^ *p* < 0.0001. **(B)** Caspase 3 activation and **(C)** DNA fragmentation in human H1975 tumor cells treated with purified Pabs from rabbits immunized with the Mv-HER3 vaccine candidate. Untreated cells and cells treated with irrelevant Pabs served as negative controls, while MitC (5 μg/mL) was used as a positive control for both assays. The results shown are representative of two independently performed experiments.

To determine if the effect of Mv-HER3-induced PAbs on these oncogenic receptors is traduced into an antitumor effect, we first evaluated their cytotoxic effect on several human tumor cell lines that express HER3, as well as HER1 and HER2 (including breast, prostate, and lung tumor cell lines). As shown in **Figure 5**, incubation with anti-HER3 sera caused a significant reduction in the viability of all evaluated human tumor cells, consistent with the relevance of HER3 in the evaluated tumor types (6–10). Treatment with anti-HER3 serum resulted in more than an 85% reduction in cell viability in the DU145, PC9ER, and H1975 cell lines. DU145 is a prostate tumor cell line resistant to androgen deprivation therapy (ADT), while both PC9ER and H1975 express the EGFR-T790M mutation that predicts resistance to first-generation anti-HER1-TKI. These results are consistent with previous reports that highlight HER3 as a relevant trigger in the acquisition of a resistance-competent phenotype in lung and, more recently, prostate carcinoma. No cell viability reduction was observed in the PC3 tumor cell line (see **Supplementary Figure S5**), which lacks HER3 expression, suggesting that viability reduction is dependent on HER3 targeting and, hence, specific.

Considering that the antigen is necessary for the formulation to specifically induce a response against HER3, sera induced by immunizations with the adjuvant mixture (without the antigen) exhibited no cytotoxic effect on cells expressing HER3 (see **Supplementary Figure S6)**.

Motivated by studies showing the connection between HER3 inhibition and inducing apoptosis in tumor cell lines (11, 32), we investigated whether the PAbs produced by the HER3-based vaccine candidate trigger markers of apoptosis in cells expressing EGFR family receptors. This could potentially explain how the direct recognition of the PAbs generated by vaccination, leading to receptor degradation, causes a cytotoxic effect on tumor cells. We used MitC, as a positive control in these assays. In **Figures 5B, C**, we observed an increase in the percentage of active caspase 3 and fragmented DNA in cells treated with the HER3-specific PAbs compared to those cells treated with the corresponding negative controls. Together, both markers suggest the induction of apoptosis by HER3-specific PAbs.

### 3.5 Mv-HER3 induced-PAbs elicit antitumor effect when passively transferred to nude mice bearing DU145 human tumors

**Figure 6:**
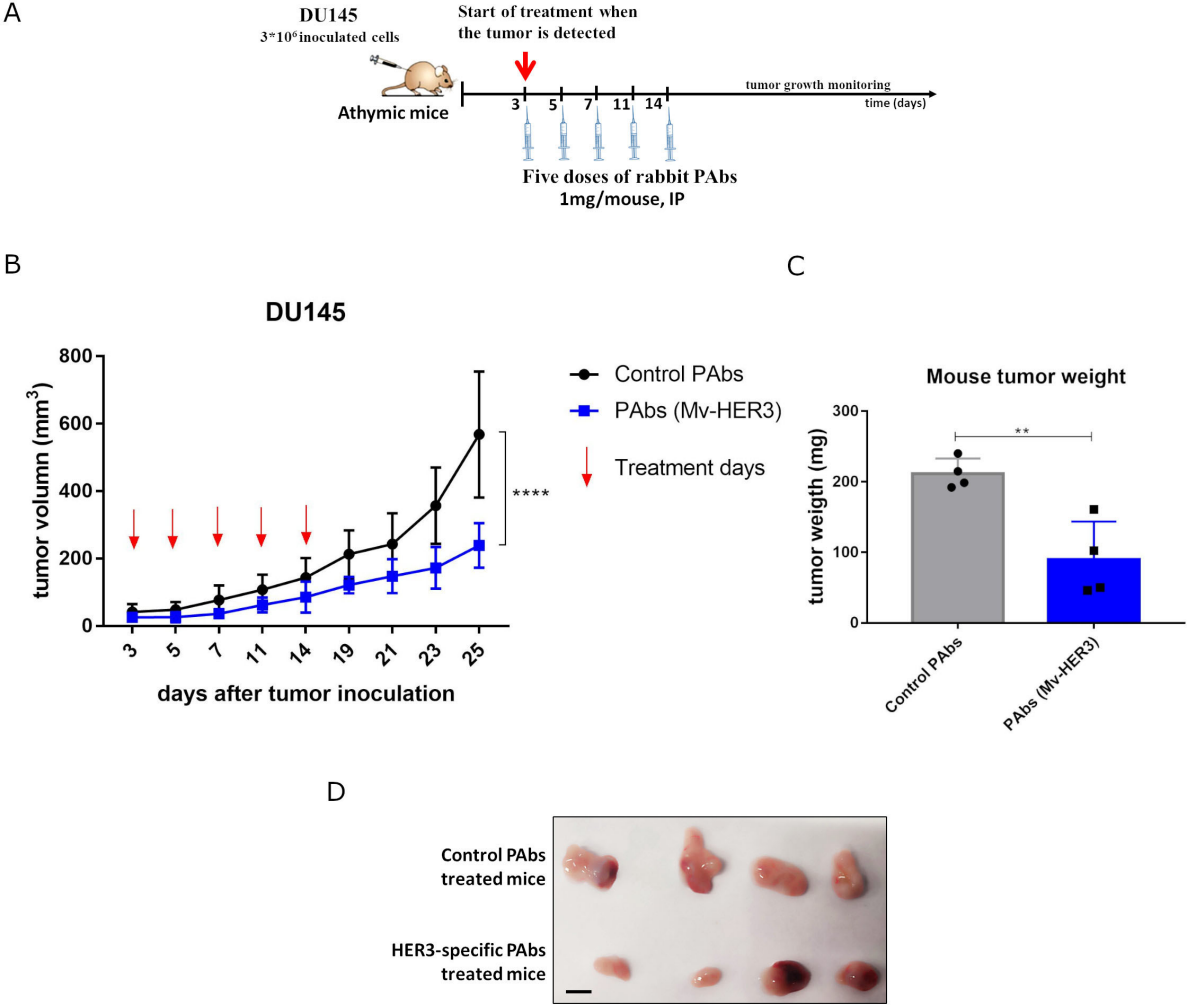
Antitumor effect of PAbs generated by immunization with the HER3-ECD-specific vaccine candidate. **(A)** The experimental scheme followed: athymic mice were challenged subcutaneously with 3 * 10^6^ cells of DU145 tumor cell line (diluted in 100 µL of PBS and injected into the right flank). Once tumors were palpable, mice were treated intraperitoneally (IP) with 1 mg of Mv-HER3-induced PAbs (from immunized rabbits). **(B)** Five doses were administered, and tumor growth kinetic curves were determined. **(C)** Average weight and **(D)** images of tumors extracted from mice treated with Mv-HER3-induced or irrelevant PAbs are shown. Scale bar, 1 cm. Tumor growth kinetic curves were compared using two-way ANOVA, while differences in means for average weight in **(C)** were analyzed using a *t*-test. Significant differences are represented as ^**^ *p* < 0.01; ^****^ *p* < 0.0001. The results shown are representative of two independently performed experiments.

To evaluate the antitumor effect of the polyclonal antibody response generated by the HER3-based vaccine candidate, IgG isotype PAbs were purified from the sera of rabbits immunized with Mv-HER3, which contained HER3-specific antibodies (see **Supplementary Figure S7**). Given that the DU145 tumor cell line was sensitive to the treatment with the PAbs generated by Mv-HER3, this tumor line was selected as a human model to evaluate the *in vivo* antitumor effect of the Mv-HER3-induced PAbs. In this context, purified PAbs were transferred to nude mice carrying tumors derived from DU145 prostate carcinoma after the tumors became detectable. Following a five-dose treatment scheme (**Figure 6A**), statistically significant differences in tumor growth were observed between mice treated with Mv-HER3-induced PAbs and those treated with PAbs purified from nonimmunized rabbits (239 mm^3^ ± 65.7 mm^3^ vs. 568.1 mm^3^ ± 187 mm^3^, *p* < 0.0001) (**Figure 6B**). This result was confirmed when tumor weights from treated and control animals were compared, as vaccination-induced PAbs significantly reduce tumor weight at the experimental endpoint (239 mm^3^ ± 65.7 mm^3^ vs. 568.1 mm^3^ ± 187 mm^3^, *p* < 0.01) (**Figures 6C, D**).

## 4 Discussion

HER3 expression or overexpression has been described in multiple epithelial tumors, including breast, ovarian, lung, colon, pancreatic, melanoma, gastric, head and neck, and even prostate cancers (15, 33–36). It is now widely accepted that HER3 overexpression is a mechanism of tumor-acquired resistance to different treatments, as this receptor provides compensatory signaling and, more importantly, drives persistent signaling through the PI3K/Akt pathway (1, 2). These peculiarities of HER3 have motivated the development of specific targeted therapies. In this context, passive therapies based on MAbs, bispecific antibodies, and MAbs conjugated to drugs stand out. However, fewer examples of active immunotherapy (specifically vaccines) targeting HER3 have been described in preclinical and clinical reports (2). Addressing HER3 from the perspective of active immunotherapy could have offer advantages over passive therapies beyond its proven safety, such as the possibility to trigger different adaptive immune effectors, induce memory, and generate a wide repertoire of antibodies against different epitopes. The latest could enhance the endocytosis and degradation of the targets, resulting in a consequent superior impact on tumor cells compared to MAbs, whether used alone or in combination (25, 26, 37).

Although active immunotherapy constitutes an alternative for the treatment of cancer, it presents the additional challenge of activating the immune system of cancer patients suppressed by the tumor itself (37). In this study, a monovalent vaccine candidate was designed based on HER3-ECD, which uses a combination of adjuvants (VSSP and Montanide) to trigger an immune response against a self-antigen, as successfully achieved previously for HER1 (28, 38). Hence, the importance in the design of cancer vaccines is not only the selection of relevant Ags to the tumor biology, such as the HER3 receptor, but also of adjuvants capable of activating the patient’s immune response. In this context, VSSP was successfully used as an adjuvant in vaccines targeting self-antigens evaluated in preclinical models and patients (28, 29, 39). In 2012, the VSSP/Montanide combination was shown to induce specific neutralizing antibodies that recognize the receptor on the membrane of tumor cells, thereby inhibiting the activation of signaling cascades (40). This adjuvant combination in a human self-antigen-based vaccine has also been shown to induce higher antibody titers compared to using these adjuvants alone (41). All of this background supports the use and combination of these adjuvants in formulating the HER3-based vaccine candidate.

Avoiding tolerance has been one of the greatest challenges in vaccinology with autoantigens, as the immune system is designed to prevent self-destructive reactions mediated by autoreactive lymphocytes (42). As part of the generation of the HER3-based vaccine candidate, this work demonstrated that the murine ErbB3 receptor, when included in the proposed adjuvant formulation, was immunogenic in mice. To our knowledge, these results constitute the first reports of the ability of a mErbB3-based candidate to break tolerance to autoantigens in autologous settings. Additionally, these results suggest that the Mv-HER3 vaccine may circumvent tolerance and induce high antibody titers in patients. The ability of the PAbs generated by the Mv-HER3 candidate to recognize the murine ErbB3 receptor allowed us to evaluate the antitumor effect of this candidate in the 3LL-R3 tumor cell line and will also allow us to extend the evaluation of the antitumor effects to several mErbB3-positive mouse models. Furthermore, the antitumor effect observed in mice immunized with both murine and human ErbB3 receptors as antigens and subsequently challenged with a metastatic tumor resistant to an anti-EGFR MAb suggests the potential of vaccination with the Mv-HER3 candidate in the context of HER3-overexpressing carcinomas that have acquired resistance to antitumor therapies.

As further evidence supporting the use of the Mv-HER3 candidate in resistant scenarios, the passive transfer of the PAbs generated by immunization with the HER3-based candidate to mice bearing human DU145 tumors inhibited tumor growth compared to control mice. Recent studies have described HER3 overexpression in prostate models resistant to ADT (9), highlighting its potential as a therapeutic target. However, this is the first report of an active immunotherapy specific for HER3, with its antitumor potential tested in a scenario of ADT resistance. Consistent with these *in vivo* findings, the largest cell viability reduction caused by vaccination-induced PAbs was observed not only in the DU145 cell line (ADT resistant) but also in H1975 and PC9ER NSCLC lines resistant to anti-HER1 TKIs. This reinforces the potential of this vaccine candidate in the context of cancer patients who have relapsed after conventional therapies.

Passive transfer of PAbs into tumor-bearing mice has been previously used by others to demonstrate the role of the humoral response induced by a vaccine in its overall antitumor effect (25). However, the contribution of the cellular response potentially induced by this vaccine to its antitumor effect cannot be ruled out, although it remains to be demonstrated. In this regard, the selected adjuvants (VSSP and Montanide) could provide the immune system with the “danger” signals necessary for activating DCs, polarizing toward a Th1 response, and stimulating specific CD8 T cells (40). Furthermore, some of the induced antibodies might indirectly activate cytotoxic CD8 lymphocytes, a phenomenon that has been previously described for immunization with recombinant adenoviral vectors encoding full-length human HER3 (Ad-HER3-FL) (43).

In addition, the present study provides some evidence for the molecular mechanisms associated with the antitumor effect of Mv-HER3-induced PAbs. Once the capacity of the PAbs induced by the HER3-based vaccine candidate to specifically recognize this receptor in tumor cells was demonstrated, it was shown that this recognition led to HER3 degradation in several tumor lines. These results agree with those reported by other authors, who proved that the recognition of a membrane receptor by specific antibodies promotes its degradation (38, 44). Given the direct connection between HER3 and resistance to cell death, the cytotoxic effect of induced PAbs on all HER3-positive tumor lines may be attributed to their ability to degrade this target. This cytotoxic effect likely occurs through the induction of apoptosis, as supported by our findings. Several studies have linked HER3 inhibition with the induction of apoptosis (11, 32). Furthermore, the degradation of HER1 and HER2 by polyclonal antibodies has also been associated with apoptosis induction (38). Considering that the HER3-specific PAbs generated by the candidate can also degrade these receptors (HER1 and HER2), it is likely that this mechanism further enhances apoptosis induction in cells treated with the generated PAbs from the HER3-based vaccine candidate.

Effector functions of MAbs have been linked to the recognition of different structural regions. Specifically, depending on the subdomain that MAbs recognize, they can block ligand binding, prevent homo- or heterodimerization, trigger internalization and degradation of the receptor, or engage the immune system in the destruction of tumor cells through antibody-dependent cell-mediated cytotoxicity (ADCC) or complement-mediated cytotoxicity (CDC) (45). Moreover, the recognition of nonoverlapping epitopes on a surface antigen by MAbs enhances antibody-receptor lattice formation, which leads to the endocytosis and degradation of these complexes (25). For vaccination-induced PAbs, specific functions have also been associated with the subdomain/s that is/are preferentially recognized (46). The PAbs induced by the Mv-HER3 candidate are capable of recognizing all four subdomains of HER3. While this polyclonality was expected given that the whole extracellular domain of HER3 was used as the antigen, this result not only explains PAbs’ potential to downregulate the target receptor but also suggests that effector functions beyond target degradation could be elicited by these PAbs. Moreover, it would be interesting to evaluate whether simultaneous recognition of multiple epitopes of HER3 by these PAbs might enhance tumor inhibition with regard to specific MAbs, which could suggest a differentiation strategy from passive therapies, as reported by Bergado et al. in 2022 (25).

The antitumor response driven by polyclonal antibodies may be better than the response induced by a specific monoclonal antibody targeting HER3 for different reasons. Several studies have consistently indicated that a combination of antibodies targeting nonoverlapping epitopes elicits superior antitumor responses (such as receptor degradation, cytotoxic effects, and tumor growth reduction) compared to individual antibodies used alone, suggesting the advantages of the polyclonal antibody response (25–27). Additionally, a study by Bergado et al. in 2022 comparing the polyclonal antibody response of a HER1/HER2 bivalent vaccine with registered monoclonal antibodies against HER1 and HER2 revealed that the combination of polyclonal antibodies against HER1/HER2 exhibited superior antitumor properties in terms of receptor degradation and cytotoxic effects compared to HER1/HER2 monoclonal antibodies, both individually and in combination (25). However, to confirm our hypothesis, future studies should address the comparison of the antitumor effects of HER3-specific PAbs with HER3-specific monoclonal antibodies (even with a mixture of them).

Remarkably, incubation of tumor cells expressing HER1, HER2, and HER3 with Mv-HER3-induced PAbs caused a reduction of HER1 and HER2 protein levels, although the PAbs did not recognize HER1 or HER2. These results suggest that vaccination-induced PAbs may be capable of degrading HER3 in the context of heterodimers with HER1 and HER2, subsequently affecting the expression of these interacting partners. Although this hypothesis needs further demonstration, it suggests the feasibility of combining HER3 as an antigen with other HER receptors (specifically oncogenic receptors HER1 and HER2) in the context of multivalent vaccines. This approach could improve cytotoxicity induction by the induced PAbs and the antitumor effect of the vaccine candidate, even in models representative of resistance. The multiantigen inhibition of HER receptors by immunotherapies was initially evaluated from the perspective of peptide vaccines (47); however, this format induces a much less diverse repertoire of PAbs compared to protein-based vaccines, resulting in fewer effector attributes.

In summary, we propose a protein-format vaccine candidate that uses HER3-DEC, combined with VSSP and Montanide as adjuvants, with the potential to induce an antitumor effect in patients with HER3-positive tumors. Our findings are consistent with and support previous reports, which propose that PAbs induced by cancer vaccines targeting oncogenic receptors promote antigen degradation and interfere with tumor cell growth *in vitro* and/or tumor progression *in vivo*. This approach could be extended to additional molecules relevant to tumor biology (38, 44).

## Data Availability

The original contributions presented in the study are included in the article/**Supplementary Material**. Further inquiries can be directed to the corresponding author.
