# Supplementary material for: Antitumor activity of PAbs generated by immunization with a novel HER3-targeting protein-based vaccine candidate in preclinical models
Source: Front Oncol. 2024 Oct 16;14:1472607. doi: 10.3389/fonc.2024.1472607 (PMC11521786; doi:10.3389/fonc.2024.1472607)
Supplement: Supplementary file 1 [file DataSheet1.pdf]

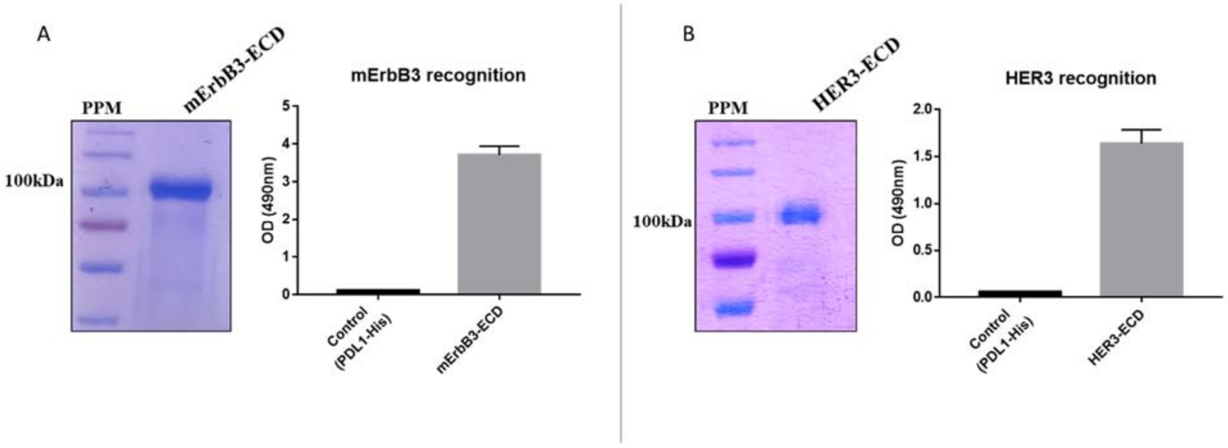

Supplementary Figure 1. Generation of the extracellular domains of the murine and human variants of the ErbB3 (mErbB3 and HER3). HEK293 cells were transiently transfected with the extracellular domains (ECDs) of ErbB3/HER3 (with a 6X-His tag) cloned in to the pCMX vector. 7 days after transfection, ECDs of murine ErbB3 **(A)** and HER3 **(B)** were purified from the supernatant by Immobilized Metal Chelate Affinity Chromatography. The concentrations and purity of both proteins were determined by measuring the UV absorbance at a wavelength of 280 nm and by SDS-polyacrylamide gel electrophoresis (SDS-PAGE) (7,5%) in reducing conditions, respectively. The SDS-PAGE are showing a band with molecular weight around the 100KDa, in agreement with the theoretical molecular weight for mErbB3 **(A)** and HER3 **(B)** purified protein. The identity of the proteins was confirmed by ELISA, for both murine ErbB3 (A) and HER3(B) proteins using commercial anti-mErbB3 and anti-HER3 monoclonal antibodies. The protein solution was 0.2µm filtered and stored at 4°C until further usage.
