# Supplementary material for: Antitumor activity of PAbs generated by immunization with a novel HER3-targeting protein-based vaccine candidate in preclinical models
Source: Front Oncol. 2024 Oct 16;14:1472607. doi: 10.3389/fonc.2024.1472607 (PMC11521786; doi:10.3389/fonc.2024.1472607)
Supplement: Supplementary file 2 [file DataSheet2.pdf]

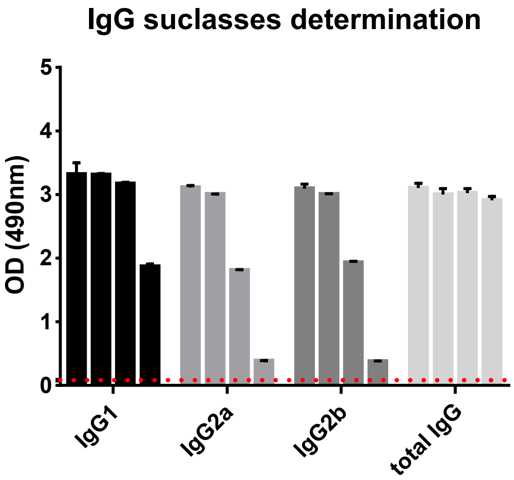

Supplementary Figure 2. IgG subclasses determination in the sera of mice immunized with the Mv-HER3 vaccine candidate. For determination of serum IgG subclasses, secondary isotype-specific biotinylated rat anti-mouse IgG1 (cat: 553441), IgG2a (cat: 553388) or IgG2b (cat: 553393) were used (PharMingen). In this experiment, ELISA plates were coated with 5µg of ECD-HER3 recombinant protein. After incubation with different dilutions ($1/10^2$, $1/10^3$, $1/10^4$ and $1/10^5$) of pooled sera from immunized mice with four doses of the vaccine candidate, we added mouse-specific IgG1, IgG2a, or IgG2b biotinylated antibodies (diluted at 1:4000), followed by HRP-conjugated Streptavidin (016-030-084, Jackson) (diluted at 1/5000). For total IgG determination was used the goat anti-mouse IgG (Fc specific)–Biotin antibody (B7401, Sigma). The graph shows the absorbance value obtained for each IgG subclass and total IgG.
