# Supplementary material for: Antitumor activity of PAbs generated by immunization with a novel HER3-targeting protein-based vaccine candidate in preclinical models
Source: Front Oncol. 2024 Oct 16;14:1472607. doi: 10.3389/fonc.2024.1472607 (PMC11521786; doi:10.3389/fonc.2024.1472607)
Supplement: Supplementary file 3 [file DataSheet3.pdf]

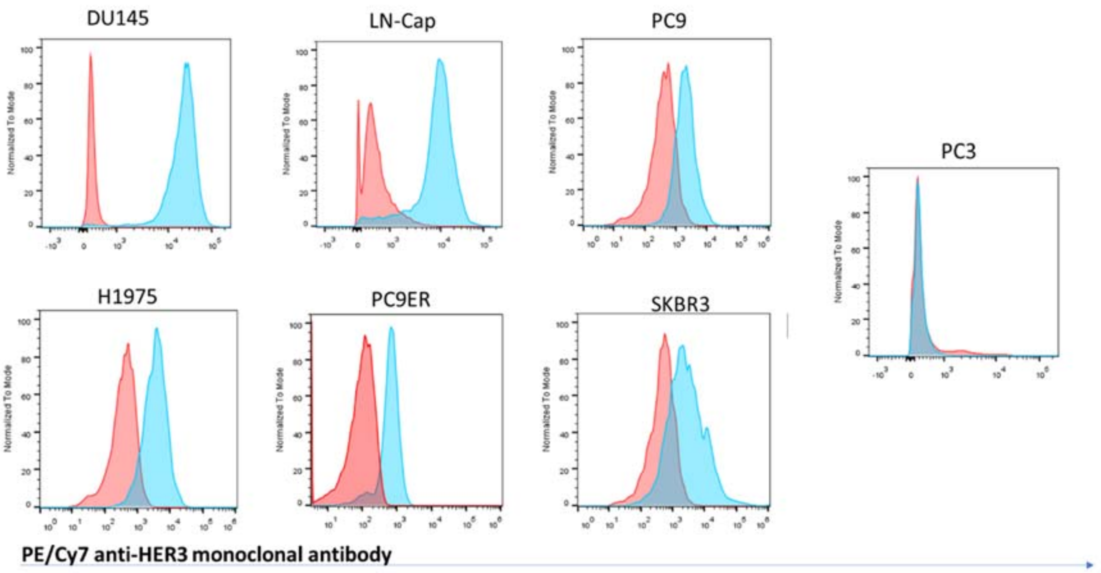

Supplementary Figure 3. Recognition of HER3 molecule in the membrane of different human tumor cell lines. DU145, LN-Cap, PC9, H1975, PC9ER, SKBR3 and PC3 cells were stained with the PE/Cy7 anti-HER3 monoclonal antibody (324709, BioLegend) Five thousand events were acquired in a Gallios cytometer (Beckman Coulter, San Jose, CA, USA) and data were processed with FlowJo 10 software. Non-stained cells are represented with the red histograms and stained cell are represented with the blue histograms
