# Supplementary material for: Antitumor activity of PAbs generated by immunization with a novel HER3-targeting protein-based vaccine candidate in preclinical models
Source: Front Oncol. 2024 Oct 16;14:1472607. doi: 10.3389/fonc.2024.1472607 (PMC11521786; doi:10.3389/fonc.2024.1472607)
Supplement: Supplementary file 4 [file DataSheet4.pdf]

A

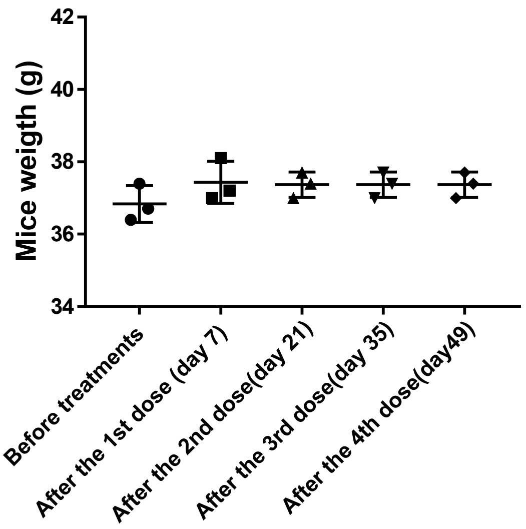
Mice weigth (g)
42
40
38
36
34
Before treatments
After the 1st dose (day 7)
After the 2nd dose(day 21)
After the 3rd dose(day 35)
After the 4th dose(day49)

B

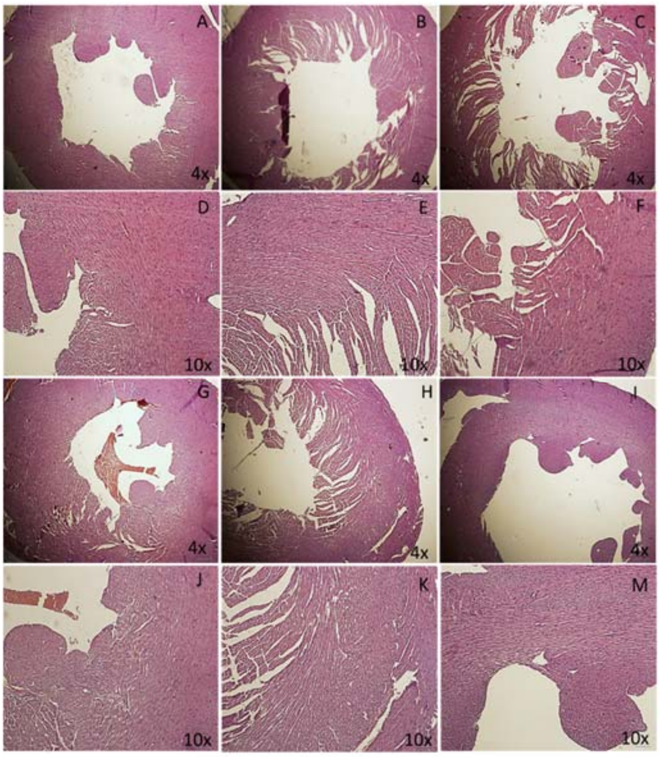
A
B
C
4x
D
E
F
10x
G
H
I
4x
J
K
M
10x

C

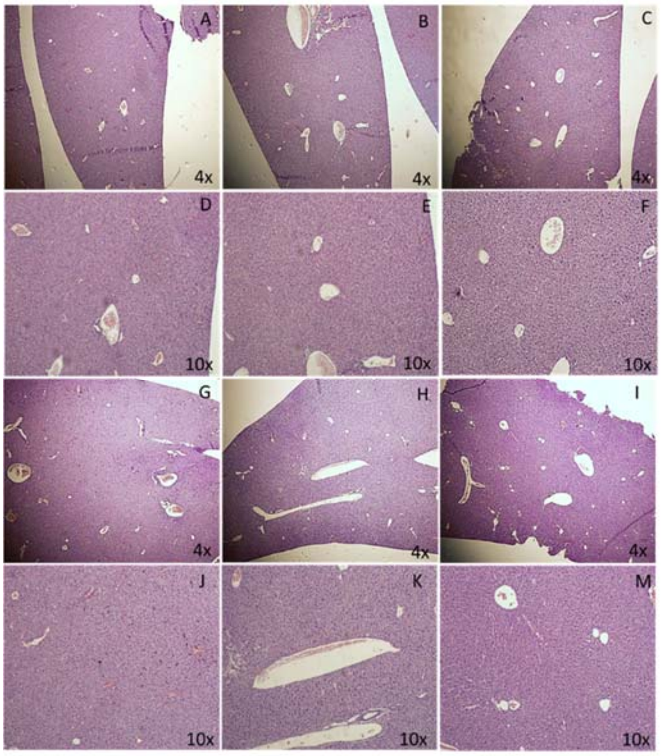
A
B
C
4x
D
E
F
10x
G
H
I
4x
J
K
M
10x

D

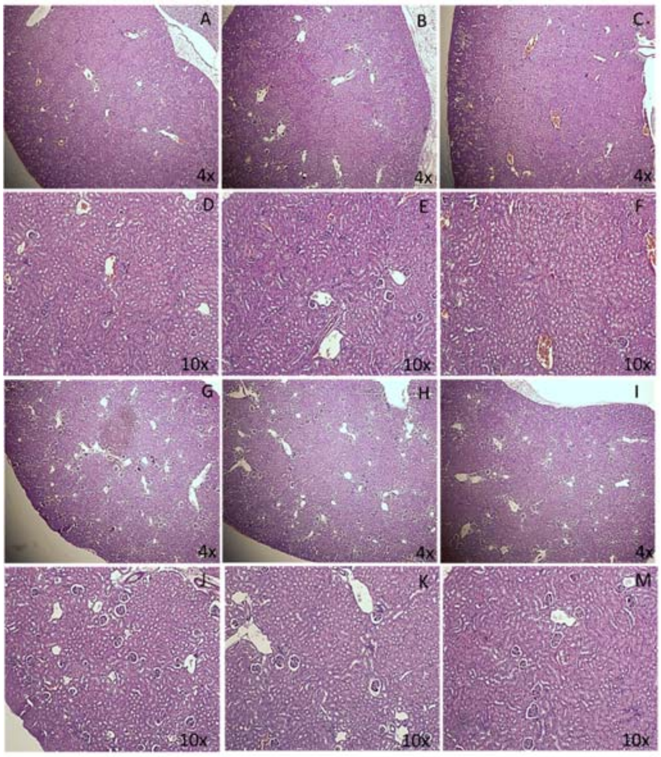
A
B
C
4x
D
E
F
10x
G
H
I
4x
J
K
M
10x

Supplementary Figure 4: Toxicological analysis of the HER3-based vaccine candidate in mice. To assess the toxicity of the Mv-HER3 vaccine candidate, a control group (images A-F, for each organ tissues) and a group receiving the Mv-HER3 candidate vaccine (images G-M, for each organ tissues) were compared (3 mice per group). The mice's weight was measured after each immunization (A). After the vaccination protocol was completed, the animals were sacrificed to analyze signs of toxicity in organs such as the heart (B), kidney (C), and liver (C). The tissue fragments of the different organs were fixed in neutral buffered formalin and processed by inclusion, and cut for the paraffin method. Hematoxylin-eosin staining was performed. The tissues from different organs were analyzed for specialists to identify the cortex, parenchyma and stroma, and the presence or absence of inflammatory lesions (edema and inflammatory infiltrate). No visible adverse effects or signs of toxicity were observed in the behavior, weight, or the histology of the analyzed organs, indicating the safety of the HER3-based vaccine candidate.
