# Supplementary material for: Antitumor activity of PAbs generated by immunization with a novel HER3-targeting protein-based vaccine candidate in preclinical models
Source: Front Oncol. 2024 Oct 16;14:1472607. doi: 10.3389/fonc.2024.1472607 (PMC11521786; doi:10.3389/fonc.2024.1472607)
Supplement: Supplementary file 5 [file DataSheet5.pdf]

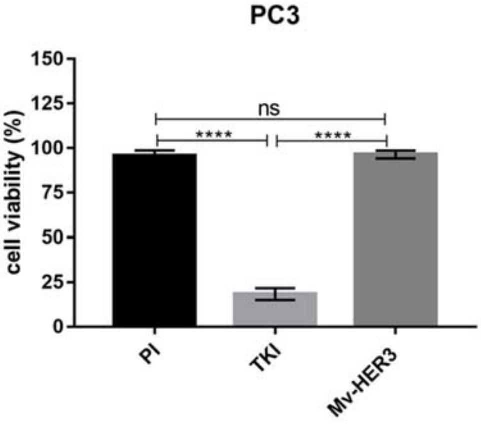

Supplementary Figure 5. Inhibition of cell viability in PC3 human tumor cell line treated with sera obtained from mice immunized with the Mv-HER3 candidate. PC3 cell line was treated for 96h with a mixture of sera obtained from mice immunized with the Mv-HER3 vaccine candidate (heat-inactivated and diluted 1:20). Cell viability was quantified by MTT assay. Pre-immune (PI) sera were used as a negative control. AG1478 TKI was used as positive controls. The graph is shown one experiment representative of two conducted. Differences among means were analyzed using one-way ANOVA and Tukey test for multiple comparisons. Significant differences among treatments are represented as ****$p < 0.0001$.
