# Supplementary material for: Antitumor activity of PAbs generated by immunization with a novel HER3-targeting protein-based vaccine candidate in preclinical models
Source: Front Oncol. 2024 Oct 16;14:1472607. doi: 10.3389/fonc.2024.1472607 (PMC11521786; doi:10.3389/fonc.2024.1472607)
Supplement: Supplementary file 6 [file DataSheet6.pdf]

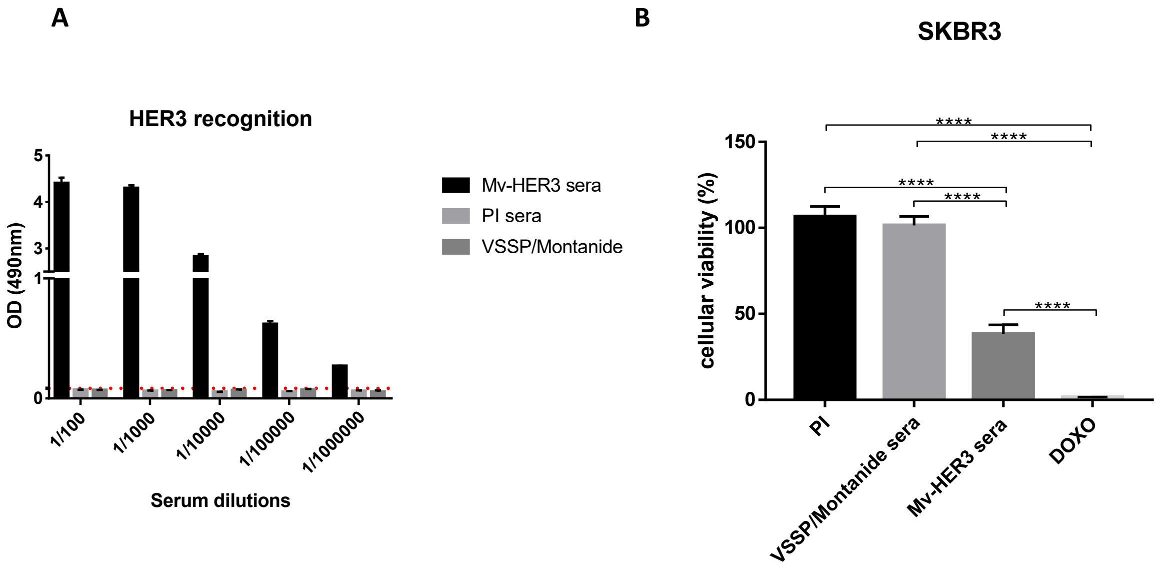

Supplementary Figure 6. HER3 recognition and cytotoxic effect of sera generated with Mv-HER3 candidate and with the VSSP/Montanide mixture immunization. **(A)** Microtiter plates (High binding, Costar, USA) were coated with 10µg/mL of HER3-ECD-His recombinant proteins in carbonate buffer, 0.1M, pH 9.6, and incubated overnight at 4°C. Then, plates were washed with 0.05% Tween 20 in PBS (Washing Buffer) and blocked for 1h at room temperature with assay buffer (4% Bovine Serum Albumin and 0.5% Tween 20, in PBS). Dilutions of sera: i- from mice immunized with the Mv-HER3 candidate (Mv-HER3), ii- from mice immunized with a formulation containing only the VSSP/Montanide adjuvant combination (VSSP/Montanide), and iii- preimmune sera (PI) were incubated for 1 h at 37°C, followed by an incubation with HRP-conjugated goat anti-mouse IgG antibody (71045, Sigma, USA) for 1h at 37°C. Finally, orto-phenylendiamine (OPD) peroxidase substrate (Sigma) was added and plates were light protection incubated for 30 min at room temperature (RT). The reaction was stopped using 10 M H_2SO_4. The optical density (OD) at 490 nm was measured using a microwell reader (Organon Teknica, Salzburg, Austria). All incubations were followed by three washing steps with washing buffer. An OD value of at least twice the value of the background control (red dashed line) was established as a criterion to consider a positive signal in the test. All samples were evaluated in triplicate. **(B)** Cell viability was assessed by MTT (M5655, Sigma-Aldrich). 5×10^3 cells/well of SKBR3 cell line were plated in 96-well plates and incubated with sera: i- from mice immunized with the Mv-HER3 candidate (Mv-HER3), ii- from mice immunized with a formulation containing only the VSSP/Montanide adjuvant combination (VSSP/Montanide), and with iii- pre-inmune sera (PI) diluted 1:20 in growth media and supplemented with 1% FBS. Previous to their addition to the cells, sera were incubated at 56°C for 30 minutes to inactivate the complement. Cells treated with pre-immune sera (pre-heated and diluted as described) were considered as negative control for the assay. After 96 h, the MTT reagent was added to the cells (1 mg/ml), and 2 h later, the formazan crystals were dissolved in DMSO. Absorbance was measured at 540 nm, and background at 630 nm was subtracted. Untreated cells were considered as maximum viability control. Doxorubicin (DOXO) (D1515, Sigma-Aldrich) was used as a general cytotoxicity induction control (10µM). Treated cell viability percentage was calculated according to the next formula: cell viability (%) = (OD treated $cell_{540nm-630nm}$)/ (OD non-treated $cell_{540nm-630nm}$) × 100. All samples were evaluated in quintuplicate. Differences among means were analyzed using one-way ANOVA and Tukey test for multiple comparisons. Significant differences among treatments are represented as ****$p < 0.0001$.
