# Supplementary material for: Antitumor activity of PAbs generated by immunization with a novel HER3-targeting protein-based vaccine candidate in preclinical models
Source: Front Oncol. 2024 Oct 16;14:1472607. doi: 10.3389/fonc.2024.1472607 (PMC11521786; doi:10.3389/fonc.2024.1472607)
Supplement: Supplementary file 7 [file DataSheet7.pdf]

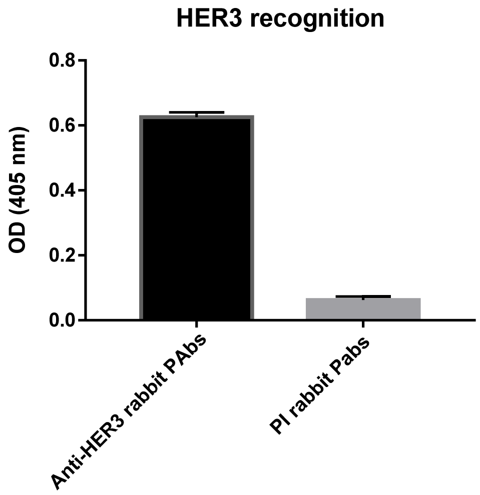

Supplementary Figure 7. Recognition of HER3-ECD of the PAbs generated in rabbits by immunization with the Mv-HER3 vaccine candidate. Microtiter plates were coated with 10 µg/ml of the extracellular domain of HER3. Then, 10µg/mL of the rabbit PAbs were applied, followed by addition of sheep anti-rabbit IgG conjugated to alkaline phosphatase. As negative control, irrelevant PAbs purified from non-immunized rabbit were applied.
